# Exploring Proof of Concept for a Novel Web-Based Self-Management Support Intervention for Polycystic Ovary Syndrome: Multimethod Study

**DOI:** 10.2196/69206

**Published:** 2026-02-17

**Authors:** Carol Percy, Andy Turner, Bethan Alper, Petra A Wark

**Affiliations:** 1 Centre for Intelligent Healthcare Coventry University Coventry United Kingdom; 2 Centre for Healthcare and Communities Coventry University Coventry United Kingdom; 3 Hope For The Community Community Interest Company Coventry United Kingdom

**Keywords:** polycystic ovary syndrome, PCOS, women’s health, self-management, peer support, web-based health intervention, psychoeducation, anxiety, depression, positive wellbeing

## Abstract

**Background:**

Polycystic ovary syndrome (PCOS) is a common chronic hormonal condition affecting 8%-13% of women and individuals assigned female at birth. Symptoms may include subfertility, menstrual, skin, and metabolic problems, with long-term health risks including diabetes and cardiovascular disease. PCOS has a significant negative impact on mental health, quality of life, and well-being. We explored proof of concept for a web-based self-management support intervention, “Hope PCOS,” designed to reduce anxiety and depression and increase positive well-being for women living with PCOS.

**Objective:**

We aim to pilot the intervention to test feasibility for web-based recruitment and delivery, acceptability, and potential to reduce anxiety and depression and increase positive well-being.

**Methods:**

Women with PCOS were recruited via social media with support from a patient advocacy charity and offered places on a 6-session cohort of the intervention. In a pre-post design, participants reported depression (Patient Health Questionnaire 9-Items), anxiety (Generalized Anxiety Disorder 7-Items), well-being (Warwick-Edinburgh Mental Wellbeing Scale), hope (SHS [State Hope Scale]), and gratitude (GQ-6 [Gratitude Questionnaire]) at baseline and 6 weeks. All participants who accessed 3 or more sessions were invited to a follow-up qualitative interview to explore user experience. Data from 8 interviews were thematically analyzed, and pre-post data were explored with descriptive statistics.

**Results:**

A total of 63 eligible women responded and were given access to the intervention. Three withdrew, leaving a baseline sample of 60, aged 20-58 (median 30, IQR 25-36) years. Further, 48 of the 60 started, of whom 46% (22/48) completed at least 3 sessions, and 29% (14/48) completed all 6. Additionally, 8 women (aged 25-38, median 29, IQR 26-35) years who completed between 3 and 6 sessions reported acceptability and experiences in exit interviews, including prioritizing self-care, developing a self-management mindset, setting motivating goals, improved mental health, self-compassion, reduced shame, openness about PCOS, preparedness for future health concerns, and continuing practice to consolidate behavior change. Furthermore, 11 women aged 25-43 (median 31, IQR 27-37) years, who completed 1-6 sessions (median 6, IQR 6-6), completed pre- and postintervention outcomes. Descriptive quantitative analysis indicated decreases in anxiety and depression and increases in hope agency, hope pathways, and gratitude. There was a meaningful (≥3 points) increase in well-being. Among patients with baseline and follow-up data, 73% (8/11) met clinical caseness for depression at baseline and 36% (4/11) post intervention.

**Conclusions:**

We explored proof of concept. Web-based recruitment and delivery online were feasible. We detected early signs of acceptability and potential benefits for anxiety, depression, and positive well-being that warrant testing in a controlled trial. Future research should assess the feasibility of a randomized controlled trial to evaluate effectiveness, acceptability, and cost-effectiveness.

## Introduction

### Background and Rationale

Polycystic ovary syndrome (PCOS) is a complex, lifelong endocrine disorder affecting women and other individuals assigned female at birth (AFAB), from menarche onward [1]. Although diagnostic criteria vary, PCOS is estimated to affect around 10% of adult women and AFAB individuals worldwide [2], and its incidence is rising in the United Kingdom [3]. Individuals with PCOS are at increased risk of several metabolic disorders, reproductive conditions, mental health issues, and sleep conditions [4-6]. PCOS has a significant adverse impact on mental health and quality of life [7,8], including higher rates of depression [9-12], anxiety, body image concerns [13], sexual dysfunction [14,15], relationship issues, and disordered eating [16-18]. While often considered a concern of the reproductive years, PCOS is lifelong, with enduring health consequences such as increased risks of type 2 diabetes, cardiovascular disease, and endometrial cancer [19-23]. The condition also has significant socioeconomic costs for affected individuals and wider society [24-27].

Recent international, evidence-based guidelines for PCOS management recommend an integrated health care approach that addresses the multifaceted nature of the condition [2]. These guidelines emphasize person-centered care, shared decision-making between patients and professionals, and advocate for patient education on cardiometabolic, dermatological, lifestyle, reproductive, and psychological aspects. Best practice also includes nonstigmatizing, weight-neutral self-management support, attentive to mental health and emotional well-being. Additionally, several countries have publicly recognized the health inequalities experienced by women in general and developed women’s health strategies to address these [28,29]. Further, the UK women’s health strategy for England [29] aims to prioritize care for gynecological conditions such as PCOS, educate and empower women and girls, and provide more support in or near patients’ homes. This includes harnessing the power of digital technology and accelerating the implementation of evidence-based digital interventions into the UK National Health Service (NHS). Person-centered, digital, self-management support interventions such as Hope PCOS have a key role to play in educating, empowering, and supporting patients with PCOS in their own homes, while complementing clinical care provided by health care professionals.

Despite recommendations from clinical guidelines and announcements of health care policy change, many people with PCOS encounter barriers to managing their condition, including limited access to specialized care or multidisciplinary treatment, lack of social support, and unmet needs for trustworthy self-management advice. High prevalence of comorbid depression and anxiety means mental health needs are often unaddressed. Psychological and behavioral interventions, especially those based on cognitive behavioral therapy, can reduce depression symptoms in PCOS, and may benefit anxiety, body image, disordered eating, and health-related quality of life [30-32]. Self-management interventions—successful in other chronic conditions such as diabetes—hold promise for PCOS, especially digital formats that are accessible and scalable. These can provide knowledge, skills, and confidence for self-management, also addressing psychological impact.

Research by the ORCHA (Organisation for the Review of Care and Health Apps) [33] has found a strong appetite for the use of digital health apps and web-based interventions across the United Kingdom, but a relative dearth of digital health literacy in those choosing apps for personal use. App users typically report basing the decision to download and use an app on how many times it has been downloaded previously, or on customer reviews, rather than checking whether the app is evidence-based or had health care provider input into its development. ORCHA suggests that individuals seeking digital health support face a largely unregulated marketplace with thousands of apps, of which as few as 20% meet basic quality standards. To address the gap for an evidence-based, digital, self-management support app for psychological well-being in PCOS, we conducted the first proof-of-concept test of an evidence-based, modular, and online intervention, Hope PCOS [34]. This intervention incorporates evidence-based PCOS-related information with hope and gratitude activities designed to increase positive well-being and improve mental health. Cultivating hope may empower individuals to cope adaptively, set and achieve meaningful goals, and maintain a positive future outlook despite health challenges [35,36]. Gratitude practices, which shift one’s focus toward positive experiences, may increase resilience and resources for chronic illness management and enhance well-being [37]. Incorporating hope and gratitude practices has the potential to reduce depression and anxiety in PCOS.

### Objectives

This proof-of-concept trial was deliberately designed as a formative, discovery-oriented investigation focused on providing descriptive evidence and understanding whether a larger, definitive trial is warranted, rather than confirming or formally assessing the impact or benefit of an intervention through formal hypothesis testing [38]. The first aim of this study was to pilot the Hope PCOS intervention to assess the feasibility of web-based delivery and its acceptability to participants. The second aim was to assess the intervention’s potential to improve anxiety, depression, and positive well-being.

## Methods

### Design

This study followed a multi-method design, primarily qualitative, with supportive, descriptive quantitative data. In a pre-post design, all participants were given access to the 6-week intervention at the same time. At baseline (T0), participants completed a web-based questionnaire confirming that they met the inclusion criteria. Please see the completed CONSORT (Consolidated Standards of Reporting Trials) Extension Pilot and Feasibility Trials Checklist [39] (Multimedia Appendix 1) for details.

Age, time since diagnosis, and secondary outcome measures were collected via web-based questionnaires at baseline, and secondary outcome measures were repeated 6 weeks after baseline, when the intervention delivery was complete (T1). At T1, all participants who completed 3 or more hope sessions were contacted and invited to take part in a follow-up semistructured telephone interview to report the acceptability of the intervention.

As this was an early proof-of-concept study, a pragmatic approach was taken to selecting the sample size. We aimed to recruit enough participants to allow for supportive but low-intensity peer interaction within the intervention cohort, which previous experience on other programs on the same digital platform suggests is likely to be at around 30-70 participants [40-42]. We initially aimed to recruit a minimum of 30 participants for this study.

### Participant Recruitment

Participant recruitment was planned for September and early October 2019, to recruit 15-30 participants. Participants were recruited from a United Kingdom community setting online. Verity, a UK PCOS advocacy organization, shared a call for research participation on their social media channels. The call included a link to this study’s website, hosted on the Qualtrics platform (Qualtrics), which is General Data Protection Regulation compliant and certified to the ISO (International Organization for Standardization) 270001 standard [43]. The website included consent and screening questions to allow adults to self-report their eligibility and complete this study’s demographics and baseline measures.

Eligibility criteria were as follows: (1) adults aged 18 years or older, (2) self-reporting a diagnosis of PCOS made by a general practitioner or a hospital specialist, (3) being based in the United Kingdom, (4) having access to the internet and a device to allow engagement with the intervention, and (5) being sufficiently fluent in English to engage with this study and intervention materials.

### Intervention

The Hope PCOS web-based self-management support intervention was delivered online via the secure internet platform provided by the social enterprise Hope for the Community (H4C) [44]. This theory-led, evidence-based self-management and peer support program was cocreated by adults living with PCOS, advocates for patients with PCOS, health care professionals, and an academic research team led by a psychologist with lived experience of PCOS. Designed for women with PCOS and other AFAB, the intervention addresses self-management needs, psychological well-being, and social support. It is underpinned by positive psychology principles, including the cultivation of hope and gratitude, and informed by the broaden-and-build theory of positive emotions to enhance emotional self-regulation, increase positive affect, and support coping with anxiety and depression. The development process is reported in detail elsewhere [34].

The online, asynchronous version evaluated in this proof-of-concept study was facilitated by trained peer supporters with lived experience of PCOS and delivered in a group format. The intervention followed a standard 6-week structure, with 1 themed educational module released on the same day each week (Multimedia Appendix 2) and designed to take about 2.5 hours to complete at the participants’ own pace. Modules included topic-specific information, audiovisual materials, cognitive-behavioral activities, and practical exercises. Two recurring elements in every session were gratitude journal entries and goal setting, with each session ending in goal setting and the following session beginning with solution-focused facilitator feedback on previous goals. Secure forums and messaging functions enabled peer-to-peer encouragement, while facilitators moderated discussions, provided supportive feedback, and monitored participation. Tailoring was achieved through open goal setting, individualized support via the forum, and flexible pacing, allowing participants to access materials at their convenience. Fidelity was planned through controlled module release schedules, peer facilitator checklists, and engagement metric monitoring. The intervention was delivered as planned. Participant adherence is described below. Please refer to the TIDieR (Template for Intervention Description and Replication) [45] checklist in supplementary files (Multimedia Appendix 3).

### Primary Outcomes

#### Overview

The primary outcome measures included adherence, engagement, and acceptability.

We calculated the proportion of participants who completed the baseline measures and subsequently logged in to the intervention platform.

#### Adherence

There is no agreement on what constitutes adherence in digital health interventions. Work on engagement in digital mental health interventions shows 7%-42% of participants typically engage in moderate use, defined as completing 40%-60% of modular, fixed-length programs or continuation of app usage after 4 weeks [46]. Participants were deemed adherent to Hope PCOS if they completed at least 3 of the 6 intervention sessions (50%).

#### Engagement

The intervention platform collects user engagement data, including the number of sessions completed per participant, and the number of gratitude entries, goals set, and comments posted.

#### Acceptability

We assessed the acceptability of the intervention by qualitative interviews at T1. All participants (n=22) who had completed at least 3 sessions were emailed and invited to take part in a private, individual audio-recorded, 30-60 minute, semistructured telephone interview 9-10 weeks post intervention. Follow-up telephone calls were made to arrange the time and date of the interview. All who accepted the email invitation (8/22) were interviewed by the H4C quality assurance manager about their perceptions and experience of the acceptability of the program. The female interviewer had a BSc and a PhD, previous experience in qualitative interviewing, and extensive experience in working with people living with long-term health conditions. To encourage open and honest disclosure from participants, the interviewer was not known to participants before the interview and was neither part of the intervention development team nor directly involved in the delivery of the intervention. She introduced herself at the beginning of the interviews, explained her role at H4C, and the purpose of the research: to explore participants’ experiences of and views on how to improve the Hope PCOS course. Interview questions included expectations and experiences of the program and any changes in mental or physical health or self-management activities attributed to participation in the program. Questions were not pilot-tested as they had been used in previous evaluations. No repeat interviews were conducted. Interview data were transcribed manually, the verbatim transcripts were checked for accuracy against the original recordings, and the audio was destroyed.

The transcripts were analyzed following the steps recommended by Braun and Clarke [47,48], which allow flexibility to use a deductive or inductive approach, or a combination of these. Reflexive thematic analysis described Braun and Clarke [47,48] supports flexible, iterative coding and theme development, and does not rely on formal coding trees as used in some other qualitative analytic traditions.

Preliminary deductive analysis of the interview data was carried out directly from the audio by the interviewer, who identified “domain summaries” of participants’ motivations for participating in the intervention, views on the appropriateness of content and delivery, any unmet expectations, and suggestions for future iterations of the intervention refinement. This preliminary analysis informed the intervention delivery team at H4C. The verbatim transcripts were then analyzed inductively by the first author, following the recommendations by Braun and Clarke [47,48] for reflexive thematic analysis. The first author, a university lecturer and experienced qualitative researcher with lived experience of PCOS, a BSc and PhD, transcribed the data manually to familiarize herself with it, reading, rereading, and noting initial impressions. She then coded the data line by line, generating initial codes that ranged from more semantic, descriptive codes, based on the manifest content of participants’ descriptions, to more conceptual codes, based on reflexive interpretation. Codes were collated into an Excel (Microsoft Corp) spreadsheet and then sorted repeatedly to search for potential themes. Themes were then identified, reviewed, and checked against corresponding coded extracts, the preliminary deductive domain summaries, and the whole dataset. Themes were then discussed with the third author, a Chartered Health Psychologist, Professor of Digital Self-Management, and experienced qualitative researcher with a BSc and PhD, before being defined and named, and the analysis was written up. This study used a mixed inductive-deductive, realist, experiential framework. Theme development was iterative and reflexive, balancing researcher and participant subjectivity with the need to rapidly inform the intervention team of potential platform and material improvements.

For reflexive thematic analysis, data saturation is not viewed as a meaningful or appropriate standard, and claims to have “reached saturation” are discouraged in favor of transparency about the reasoning behind sample size and the depth of analysis [49]. As this was an unfunded study without compensation for participation, to avoid burdening participants, member checking of transcripts and analysis was not used. Please see the completed COREQ (Consolidated Criteria for Reporting Qualitative Studies) checklist [50] in supplementary files (Multimedia Appendix 4).

### Secondary Outcomes

The secondary outcomes chosen for this study were informed by the core outcomes set for PCOS research [51,52], and by the researchers’ aim to test whether the intervention produced changes in the underpinning constructs of hope and gratitude. This was to establish proof of concept only, not to test hypotheses or the magnitude of any changes. Participants completed questionnaires at T0 (baseline) and T1 (6 weeks later, when the intervention ended).

The Patient Health Questionnaire (PHQ-9) [53] is a 9-item scale, with items measuring symptoms of depression. Items ask participants to state how much in the last 2 weeks they have been bothered by problems, including, for example, “little interest or pleasure in doing things” and “feeling down, depressed, or hopeless.” Each of the 9 items has responses ranging from 0 to 3 (0=not at all, 1=several days, 2=more than half the days, and 3=nearly every day), leading to a total score between 0 and 27. Higher scores indicate greater severity of depression. The PHQ-9 has good internal consistency (Cronbach α=0.89). Scores of 10 or more are regarded as indicating the clinical range for depression, so participants scoring 10 or more were categorized as depressed for this study. Item 9 of the PHQ-9, “thoughts that you would be better off dead, or of hurting yourself in some way,” is used to screen for suicidal ideation and suicide risk.

The Generalized Anxiety Disorder Scale (GAD-7) [54] is a 7-item scale measuring symptoms of generalized anxiety disorder. Items ask participants to state how much in the last 2 weeks they have been bothered by problems, including, for example, “feeling nervous, anxious or on edge” and “not being able to stop or control worrying.” Each of the 7 items has responses ranging from 0 to 3 (0=not at all, 1=several days, 2=more than half the days, and 3=nearly every day), leading to a total score of 0 to 21. Higher scores indicate greater anxiety. GAD-7 has good internal consistency (Cronbach α=0.92). Scores of 8 or more are regarded as indicating the clinical range for anxiety, so participants scoring 8 or more were categorized as having anxiety for this study.

The Warwick-Edinburgh Mental Wellbeing Scale (WEMWBS) [55] is a 14-item scale measuring positive mental well-being in adults. Items ask participants to state how much in the last 2 weeks they experienced certain (positive) feelings and thoughts, including, for example, “I’ve been feeling optimistic about the future” and “I’ve been dealing with problems well.” Each of the 14 items has responses ranging from 1 to 5 (1=none of the time, 2=rarely, 3=some of the time, 4=often, and 5=all of the time), leading to a total positive mental well-being score ranging from 14 to 70. Higher scores represent greater positive mental well-being. The WEMWBS has good internal consistency (Cronbach α=0.91). A change of 3 or more is regarded as a meaningful change in positive well-being [56].

We administered the GAD-7, PHQ-9, and WEMWBS at 2 timepoints—immediately before and after the 6-week intervention—using the standard 2-week recall period each time, in line with best practice and the questionnaires’ validated use. This approach was chosen to preserve the tools’ established reliability and validity, as altering the recall period or increasing the frequency of assessment might compromise data quality or increase participant burden. By anchoring assessments to the periods immediately preceding and following the intervention, we were able to capture recent anxiety, depression, and well-being status, and any changes in these, without overburdening participants.

The GQ-6 (Gratitude Questionnaire) is a 6-item scale measuring the disposition to experience gratitude [57]. Each of the 6 items has responses ranging from 1 to 7 (1=strongly disagree and 7=strongly agree), leading to a total gratitude score ranging from 6-42, with higher scores representing higher levels of gratitude. Items ask participants to state how much they agree with 3 positively worded statements about gratitude, including, for example, “I have so much in life to be thankful for,” and with 3 negatively worded (reverse coded) statements about gratitude including, for example, “when I look at the world I don’t see much to be grateful for.” The GQ-6 has good internal consistency (Cronbach α=0.76 to 0.84) [57,58].

The SHS (State Hope Scale) is a 6-item scale measuring goal-directed determination and the sense of having pathways to successfully meet one’s goals [59]. The items ask participants to select a number that describes how they think about themselves in relation to 6 statements about goal-oriented hope. Three of the statements relate to “hope agency,” a sense of oneself as being determined and motivated to achieve one’s goals, for example, “at the present time, I am energetically pursuing my goals.” Three of the statements relate to “hope pathways,” the sense of having pathways to successfully meet one’s goals, for example, “I can think of many ways to reach my current goals.” Each of the 6 items has responses ranging from 1-8 (1=definitely false and 8=definitely true), leading to a total hope score ranging from 6-48, with higher scores representing higher levels of hope. Pathways subscale scores range from 3-24, with higher scores indicating higher levels of pathways thinking. Agency subscale scores range from 3-24, with higher scores indicating higher levels of agency thinking.The SHS has good internal consistency (Cronbach α=0.93).

All quantitative data were collected via web-based questionnaires administered using Qualtrics Survey Software.

### Analysis

Descriptive analysis of quantitative data was conducted using IBM SPSS 26 (IBM Corp, released 2019). The analyses involved tabulated summaries of primary outcomes and, for secondary outcomes, pre- and post intervention, using medians and IQRs.

As a proof-of-concept study, this study was not powered to perform inferential statistical analyses. Pre- and postprogram medians and IQRs for scores on outcome measures are reported to indicate the potential to reduce depression and anxiety and increase positive well-being.

### Safety Considerations

Individuals scoring 2 or above on PHQ-9 question 9 (used to screen for suicidal ideation) were contacted to clarify that this study did not offer psychotherapy and were advised to seek professional help. They were encouraged to consult their general practitioner and given contact details for mental health agencies. Those wishing to continue were granted access to the program and trial. The digital platform includes links to suicide prevention advice and information on accessing the UK’s primary mental health care services.

### Ethical Considerations

This study received clearance from the Coventry University Research and Ethics Governance Committee (approval P93500). Participants visited this study’s website, read a participant information sheet, and ticked a digital informed consent statement before completing each web-based survey. They completed a written informed consent form before participating in the qualitative interview. Interview transcripts were anonymized for analysis, and participants are referred to by participant number, for example [P1]. No compensation was offered to any participants.

## Results

Recruitment started October 1, 2019, and ran on to October 7, 2019, via Twitter (subsequently rebranded X; the first author’s account) and Facebook (Verity PCOS’s account). A total of 63 participants responded to the social media invitation, completing the web-based consent and baseline measures.

### Descriptive Data

#### Overview

A total of 63 women completed the baseline questionnaires.

**Figure 1:**
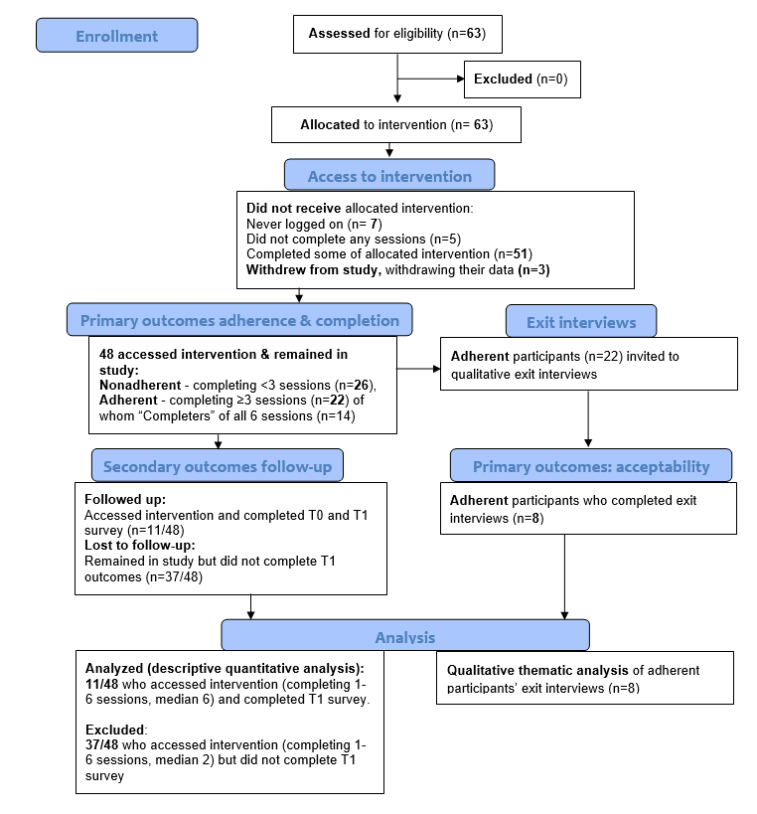
CONSORT flow diagram showing flow through proof-of-concept study for adults with PCOS in the online 6-week Hope PCOS self-management intervention. CONSORT: Consolidated Standards of Reporting Trials; PCOS: polycystic ovary syndrome.

Participant characteristics are shown below (Table 1), and participant flow through this study is shown in Figure 1.

**Table 1:** Participant demographic information at baseline for adults with PCOS^a^ who accessed the online 6-week self-management intervention and completed follow-up measures or interviews in a multi-method proof of concept study.

Characteristics	Sample at enrollment (N=60)^b^	Accessed intervention and completed baseline and postprogram questionnaire (n=11)	Completed postprogram qualitative interview (n=8)
Age (years), median (IQR)	30 (25-36)	31 (27-37)	29 (26-32)
Years since diagnosis, median (IQR)	7 (3-17)	12 (4-23)	7 (3-14)
Baseline depression caseness^c^ PHQ-9^d^, n (%)	42 (70)	8 (73)	5 (63)
Baseline anxiety GAD-7^e^, caseness^f^, n (%)	33 (55)	5 (45)	6 (75)

^a^PCOS: polycystic ovary syndrome.

^b^Excludes n=3 who withdrew from this study and whose data were withdrawn.

^c^Defined by Patient Health Questionnaire 9-Items threshold for depression.

^d^PHQ-9: Patient Health Questionnaire 9-Items.

^e^GAD-7: Generalized Anxiety Disorder 7-Items.

^f^Defined by Generalized Anxiety Disorder 7-Items threshold for anxiety.

#### Withdrawals

Three women withdrew from this study at weeks 3 (n=1) and 6 (n=2), respectively. As per the procedures in this study’s ethical approval, their data were all withdrawn and destroyed, leaving a sample of 60. For those who withdrew, 2 reported experiencing life events that made them too busy to commit to study participation, and 1 reported disliking the positive mental health focus of the intervention, stating that it was “not her cup of tea at all.” As per the data protection section in our approved participant information sheet, withdrawn participants’ personally identifiable information, age, time since diagnosis, and secondary outcome data were removed from this study’s records.

The 60 women who completed the baseline measures at enrollment and whose data remained in this study were aged 20-58 (median 30, IQR 25-36)) years. Time since diagnosis ranged from 0 to 38 (median 7, IQR 3-17)) years.

PHQ-9 scores indicated high baseline levels of depression symptoms, with 42/60 (70%) participants scoring at or above the level indicative of clinical depression. GAD-7 scores indicated high baseline levels of anxiety symptoms, with 33/60 (55%) scoring at or above the level indicative of clinical anxiety.

A total of 11 women aged 25-43 (median 31, IQR 27-37)) years, all of whom had completed 1-6 (median 6, IQR 6-6) intervention sessions, completed both pre- and postintervention outcomes. Their time since diagnosis ranged from 3 to 25 (median 12, IQR 4-23)) years.

Further, 22 “adherent” women who had completed at least 3 hope sessions were invited to an exit interview. Furthermore, 8 agreed and were interviewed. They were aged between 25 and 38 (median 29, IQR 26-32) years and had been diagnosed between 3 and 18 (median 7, IQR 3-14) years ago.

#### Harms

At baseline, 3 participants scored 2 or above on PHQ-9 item 9, indicating suicidal ideation, and were contacted as per the safety protocol. All chose to proceed with this study. No participants scored 2 or above in PHQ-9 item 9 at the T1 follow-up.

### Primary Outcomes

#### Adherence and Engagement

Of the sample of 60 at baseline and remaining in this study, 7 (7/60, 12%) never logged on to the intervention, and 5 (5/60, 8%) completed no intervention sessions. Further, 48 women who completed the baseline measures went on to complete at least part of the intervention (48/60, 80%), of whom 22/48 (46%) met the adherence definition by completing at least 3 intervention sessions, and 14/48 (29%) completed all 6 sessions.

Participants posted 256 comments and gave 157 likes of their peers’ posts, recorded 93 gratitude entries, and set 71 self-management goals, though use of the platform’s features varied widely. Adherent participants posted 0-11 comments per session (mean 1.7, SD 2.4), 0-8.3 likes of peers’ posts per session (mean 1.1, SD 2.2), recording 0-3.3 gratitude entries per session (mean 0.7, SD 0.9), and setting 0-1 goals per session (mean 0.5, SD 0.3). Nonadherent participants posted 0-2.5 comments per session (mean 0.75, SD 0.8), 0-4 likes of peers’ posts per session (mean 0.3, SD 0.8), recording 0-1 gratitude entries per session (mean 0.3, SD 0.4), and setting 0-1 goals per session (mean 0.3, SD 0.4). As a group, adherent participants posted more comments and likes of peers’ posts for each session they completed, as well as more gratitude entries and goals per session.

#### Acceptability

The 8 adherent participants who were interviewed had completed between 3 and 6 intervention sessions each (median 6, IQR 6-6). They described the intervention as acceptable and having met or exceeded expectations, with some critical feedback. All who were interviewed reported experiencing some changes in self-management behavior as a result of taking part. Themes derived from the thematic analysis are presented below (Table 2).

**Table 2:** Summary of qualitative interview themes for adults with PCOS^a^ who were adherent to the online, 6-week, self-management intervention in the multimethod proof-of-concept study.

Theme	Summary
Prioritizing self-care	Self-care is important and not selfish
Developing a self-management mindset	PCOS is valid but not dominant; openness, pacing, and gradual activity
Setting more manageable, realistic, and motivating goals	Small steps, facilitator support, and lasting change, even with low engagement
Improved mental health: being able to experience and cope with a wider range of emotions, both positive and negative	More self-awareness, acceptance, and confidence; and peer support benefits
Increased self-compassion and reduced shame	Compassion focus and peer support normalized distress
Greater openness about PCOS	Selective or trusted disclosure, sharing to help others, and advocacy
Noticing physical changes	Better sleep, more diet or exercise motivation, and return of periods
Being prepared for future health concerns	Conception planning, proactive information-seeking, and ongoing self-care
Continuing practice to consolidate or extend what was learned from the program	Ongoing skill use and lifestyle change, and long-term commitment
Experience of content, tools, and format	Concise, accurate content; ongoing resource; and personal relevance varies Gratitude aided positive focus, balance, accept bad days; and sustained use Peer facilitation aided clear or realistic goals, accountability, and overcame sharing hesitation Peer support aided motivation, connection; flexible engagement; and desire more interaction
Suggestions for future iterations of the program	Privacy options for goal sharing Extended facilitator availability Integrate into NHS^b^ care

^a^PCOS: polycystic ovary syndrome.

^b^NHS: National Health Service.

#### Prioritizing Self-Care

Taking part in the intervention allowed some participants to prioritize care for themselves and their own health. For example, P16 described participating as a process of learning and permitting herself to prioritize her own needs.

Learning to you know respect and love myself and put myself first is not a selfish thing

P16, completed 6 sessions

She emphasized that looking after her own health was not selfish. That she needed to state this reflects a difficulty in attending to her own health needs without being (or being seen as) selfish or self-centered. Another participant experienced the intervention as an opportunity to assess her own self-management needs.

It’s been encouraging me a lot more to want to think about… me (laughs) me, some of my personal needs

P27, completed 6 sessions

She laughs as she repeats “me,” again, emphasizing the difficulty in prioritizing self-care.

#### Developing a Self-Management Mindset

Several participants described changes to their way of thinking about their PCOS or responding to its challenges as a result of the intervention. These changes reflect a realization that PCOS is a valid, long-term health condition that needs to be monitored but does not necessarily have to dominate life. For example, P44 described how taking part in PCOS Hope had helped validate her health concerns and encouraged her to acknowledge these to her employer, without having to offer other explanations for illness.

I sort of feel a bit validated by my experience to the point where if I have to phone in sick because of reasons relating to my PCOS I say exactly why … instead of another reason

P44, completed 5 sessions

That P44 had previously felt her condition might not be regarded as valid seems to have discouraged her from disclosing it in her workplace, so feeling validated had enabled her to be more open about her need for sickness absence. Since taking part in the intervention, P21 had started actively monitoring her symptoms in order to recognize times when flare-ups might occur, using this to plan and pace her work accordingly.

I keep a symptoms diary which kinda helps me think to say well that potentially could be the worst day so I can’t do anything that day … don’t overcrowd your diary … allow myself permission to do that which was huge.

P21, completed 6 sessions

Pacing, often recommended by health professionals for long-term health conditions, was something that P21 felt allowed to do, having taken part in the PCOS self-management intervention.

For some participants, the self-management support provided by the program enabled them to obtain a sense of keeping PCOS in its place. For example, P26 described moving away from having her life on hold toward taking steps to become more active, in a stepwise and manageable way:

It’s actually made me think … about putting life on hold and things like that. I’ve actually started to do a little bit more so if it’s a bit more yoga and I decide to do little bits and pieces that I know that my body can handle just to get myself a little bit more active again.

P26, completed 6 sessions

The self-management mindset developed by these participants was one in which PCOS was more recognized and accepted, while not necessarily preventing active steps to continue important activities such as work and physical activity.

#### Setting More Manageable, Realistic, and Motivating Goals

Weekly goal setting and solution-focused feedback were mentioned as valuable by all participants interviewed. For example, P14, who set and shared 5 goals during the program, valued the small stepwise approach to setting manageable goals, which motivated her to keep making changes:

Just kind of the motivation to keep doing small steps … and keep setting small goals in my week … to give me a bit of focus and to keep a little bit of momentum.

P14, completed 6 sessions

The repetition of the goal setting throughout the program seems to have helped P14 maintain focus on and actively engage with her self-management. The peer facilitators’ support with goal setting was reported by P21 as particularly helpful:

At the beginning I was setting really unrealistic goals so it was really nice to have someone coming back … feedback to try to make them more manageable … it was just really helpful for me to see what I can do and that healthy expectations and when I need to draw back on unhealthy expectations.

P21

The peer facilitators’ support (using motivational interviewing skills, adjusting goal difficulty, goal specificity, assessing goal commitment, and giving solution-focused feedback) had helped P21 stay engaged with goal setting throughout the program, not giving up or becoming discouraged by missing targets that may have been unrealistic at first.

The interviewed participants who engaged less with the program and set fewer goals, still reported having gained some benefit from the goal-setting process. For example, P45, who set 2 goals in total, felt the process had had a lasting impact, and had connected with other self-management learning she had done:

The goal setting thing was actually the most useful part of the whole project and something that I’ve carried on. I think it’s sort of joined up with something else that I’d listened to about goal setting and rewarding yourself for things and so I’ve been working on that to help me improve my exercise basically.

P45, completed 3 sessions

Despite having limited engagement with goal setting during the period of the intervention, P45 continued using goal setting to increase physical activity 9-10 weeks after completing the program.

#### Improved Mental Health: Being Able to Experience and Cope With a Wider Range of Emotions, Both Positive and Negative

Several participants reported feeling their mental health had improved and that they felt this was related to attending the program. Some attributed this to being more self-aware, mindful, or accepting that they experienced distress, feeling more supported, or noticing positive aspects of life despite PCOS. For example, P16 reported feeling more in tune with her emotions and recognizing that, despite inevitable challenges, she was hopeful and more self-confident after taking part in the intervention:

I’ve got a long way to go but it doesn’t feel like like a kind of treacherous path it feels like ok you’re gonna hit stumbling blocks but you will get through it. … I’m a lot more positive and self-assured and self-confident than I was perhaps before I started the programme definitely … the programme made me tune in to more to myself …

P16

Acknowledging distress and tuning in to emotional experiences was described as part of the way through “stumbling blocks” and challenges. This was echoed by P26, who emphasized how the program has given her permission to recognize her own feelings of distress when faced by the challenges of PCOS:

Saying you know you are you are allowed to feel these things. And I think for me that was one of the biggest things.

P26

Clearly, for P26, having difficult emotions validated and validating her own experience was a major impact of the intervention. P44, who participated 12 times in group discussions, found the peer group format of delivery beneficial in providing social support and improving her mental well-being:

That definitely helped me feel like my mental health was being a bit more supported and yeah I’d say that I’m probably mentally in a better place.

P44

Not all participants found the peer group sharing helpful for their well-being. P45, who had scored as moderately severe for depression symptoms at baseline and experienced a traumatic life event during the intervention period, found the shared gratitude activities particularly difficult, especially after reading other participants’ gratitude posts:

It’s difficult when the doctor can’t think of anything to suggest and the fertility clinic can’t think of anything to suggest and then you’re sort of faced with this website and its saying all things for it to be hopeful for and you’re like oh… I think reading other people’s (gratitude posts) just made me even more down because it was like… we’re really scraping the barrel if this is the kind of stuff … (laughs). I don’t know it just made me feel even more hopeless. …

P45

This highlights a potential difficulty with positive psychology interventions and with peer group support, in that some participants may find positive psychology activities cloying or alienating, and not all participants will experience comparisons within the group as therapeutic. Despite this, P45 did conclude that there was some benefit to practicing gratitude activities herself:

The gratitude thing I think even though I found it really hard I think it, it did shift something in my brain.

P45

#### Increased Self-Compassion and Reduced Shame

Some participants reported feeling more compassionate toward themselves and the emotional distress they experienced. For some, this meant learning to practice self-compassion and reduce self-criticism. For example, P16 described the program as helping her recognize that negative thinking can be part of life, not something to judge or punish herself for:

I’m a bit of a perfectionist and I tend to also spiral into the negatives … having the programme that’s helped … the compassion focused reminding yourself you are human … that actually that’s ok its life.

P16

The peer group setting and group dynamics of the program had helped temper P18’s tendency to be self-critical:

It was a bit more of a caring environment … rather than letting me self-criticise.

P18, completed 6 sessions

Both these participants had found a shift from self-criticism toward self-compassion helpful for their mental health.

The self-compassion therapy activities in the program encouraged participants to acknowledge and not to judge their own emotional distress, reducing embarrassment and shame. For example, P14 found the program helped her appreciate how common emotional distress is in PCOS, and to feel less ashamed about her own feelings:

I feel a little bit happier about the struggles between PCOS and mental health whereas I felt a bit ashamed and kind of confused about before.

P14

Likewise, P21 reported a reduction in shame when she realized that she was not to blame for her PCOS symptoms or her associated emotional distress:

It was taking away for me the embarrassment of it and the shame that came from it and just understanding that actually it wasn’t my fault.

P21

#### Greater Openness About PCOS

Associated with a reduction in self-criticism and shame was a greater willingness for some participants to share their diagnosis with others. For some, this was a small-scale change, perhaps sharing with only a few trusted people. For others, there was an openness to sharing the diagnosis in the workplace or wider social networks. P44 reported being a little more willing to share information about her condition:

It probably has helped me to be slightly more open about my PCOS.

P44

Since taking part in the intervention, P26 had been disclosing her diagnosis at work:

I’ve probably been much more open with work colleagues.

P26

P27 was disclosing selectively and also making decisions about how disclosure to trusted social networks might get her the support she needed most:

I’m thinking probably a lot more about the networks I’m keeping and the ones that are the most supportive to me.

P27

Greater openness was associated with obtaining support for some participants, and for some, with being empowered to help others with PCOS. P16 felt empowered to share her diagnosis, and some information and tools from the program, with both a friend and her own partner:

I’ve got a friend who who’s got PCOS … I’m able to maybe offer better advice than I was before. … so in that sense it helps other people --- my partner and I … it really helped to read it to him as I was doing it.

P16

P16’s experience shows the potential for the intervention to help individuals beyond the participants themselves. This scope was broadened further by participants who decided to engage more with activism as a consequence of attending the intervention. For example, P18 described stepping up her involvement with a PCOS advocacy group and writing to a high-ranking health official to ask for change:

I’ve engaged more with Verity … I’ve also been a lot more outspoken about it … some of us are planning on sending emails to the women’s minister demanding more research.

P18

#### Noticing Physical Health Changes

Although physical health outcomes were not recorded as outcome measures in this pilot study, some participants interviewed described noticing changes in their physical health that they attributed to taking part in the intervention. For example, P26 reported improvements in sleep:

I’ve started reading mindfulness sort of activities …coming out from the programme … it’s helped with quality of sleep.

P26

P16 felt motivated to make changes to her diet and do more physical activity to manage her PCOS:

I’d already started changing to a PCOS diet and exercise and things and I had lost quite a bit of weight … it helped keep me motivated …

P16

For P27, shifting her focus to prioritize her own self-care had seen her periods return:

It’s encouraging me to think more about what’s good for my body again. Thinking about my self-care, now I’m thinking about myself more my periods have actually, they’ve actually come back. Which is like hallelujah!

P27

For P27, this change was associated with taking care of her body and prioritizing what it needed.

#### Being Prepared for Future Health Concerns

Several participants reported feeling better prepared to deal with future health concerns associated with PCOS as they arose. These might be changes related to life stage, changing family circumstances, or changes to treatment options. For example, P16 felt information gained on the program would help prepare her and her partner for future conception:

We’re getting married … the next step is children … some of the information that was in there … that’s been really helpful for both of us …

P16

P18 felt she was “armed” and empowered to proactively seek out new information about medication and treatment options in the future.

I’m armed with a little bit more information so I’m hoping as medications and things change, I will bear it in mind going forward to be proactive in keeping it that way.

P18

P18’s use of the metaphor “armed” suggests an anticipated battle for which she must continue to be prepared. P27 was also encouraged to stay informed and ready to cope with future health issues:

It’s just been another way to encourage me more to ensure that I’m well informed in the areas for myself really and my future health …

P27

#### Continuing Practice: To Consolidate or Extend What Was Learned From the Program

At the time of the interview (9-10 weeks post intervention), several participants emphasized that their use of what they had learned in the program was ongoing, and they would continue to work on it for the foreseeable future. For example, P26 was still thinking about program materials and applying changes in her everyday life:

Even now however many weeks it is away from when we finished it’s still something that I’m still processing and going through and making those changes and sort of noticing how its changing my life for me … there’s still things that I can still work on.

P26

P44 also described the “work” of self-management as ongoing, and something that required a long-term commitment of effort:

A lot of the mental health support aspects of it … I can see that having a longer-term impact – it’s something that you need to constantly work on and they’ll always feature as sort of part of your self-care … things are always a work in progress really.

P44

P14 was also motivated to keep practicing skills learned in the program to maintain and enhance her well-being:

I’m still motivated to try and work on both kind of the physical and the mental health aspects. And that’s not that’s not something that I’m going to stop any time soon.

P14

#### Experience of Content, Tools, and Format

As part of the ongoing cocreation process, participants were asked what they liked and disliked about the program content, activities, tools, and delivery format, including any recommendations for modifications to future iterations of the intervention.

Most participants interviewed reported that the evidence-based informational content presented in an online multimedia format was easy to understand and intuitive to navigate, helping them to be better informed and understand PCOS more. P26 contrasted the condensed and accessible format of the program materials to a “bombardment of books” she had encountered elsewhere:

There was a lot of information in there that… some of it I knew a lot of it I already knew but it was nice to have it condensed and simplified rather than being in a bombardment of books that three or four hundred pages long and you’ve got loads of information to take in.

P16

P18 contrasted the accuracy of program materials with unfiltered material often found on the internet:

Accessing that information, which I know is accurate as well cos that’s another downside of “Doctor Google” you don’t know what you’re gonna come across. I know that that (programme information) is backed and up to date and everything so that’s a reassuring resource.

P18

Further, 2 participants described the information acquired in the program as a “bank” they could return to later as required, for example, P16 had saved recommended links to her internet history to consult in the future:

Having that knowledge bank in my mind and having some of the things saved on my internet history so I could go back to them helped me to seek the help and seek the different things that I needed in order to improve my life in other areas as well.

P16

Most participants interviewed reported finding the information component of the program helpful or personally relevant, but P45 did not find anything new of interest:

Quite a lot of the sort of PCOS related stuff was stuff I already knew so working through that wasn’t helpful … I’ve had my diagnosis for quite a long time so I, if I’d been new to it it would have been helpful but at this point it wasn’t.

P45

Participants interviewed described positive experiences of using the self-management tools provided. The gratitude activities were described as important in maintaining emotional well-being. For example, P26 described focusing beyond the negative:

It probably helps with the mindset thing is thinking about the things that are that are good … that are good in life and not necessarily focusing on all the … you know the negative things that are going on.

P26

P27 had continued to use the gratitude journaling activity after the intervention period and found it a useful response to distress:

Actually doing them it was quite rewarding and it just gives them that positive that positivity again so even if things are a little bit shaky during the day you’re always remembering the positives which I suppose is a balance.

P27

P16 also referred to using the gratitude activities to maintain emotional balance:

I really liked having that kind of try and find something positive in your day. As I say some days were awful and you just can’t comment and that’s fine and that’s life. It was similar with one of the talks they had a video about positivity thing cos again I’d read (inaudible) book about face the fear and do it anyway and everything in that book sort of was like everything has to be positive in life and sometimes it isn’t. Whereas the video and having the gratitude enabled you to kind of get the right balance that there are positives even on a bad day but some days are just bad and that’s ok to accept when it is bad. And that really helped so yeah. It gave some balance.

P16

What P16 describes here is using gratitude to maintain perspective on both positive and negative emotions, rather than feeling pressure to maintain a relentlessly positive outlook—an important distinction that is embodied in gratitude interventions in chronic health conditions.

The solution-focused goal-setting support and feedback provided by the peer facilitators were valued, particularly for helping participants to set and adjust goals to make them both challenging and achievable, and in providing accountability that motivated participants to persist. For example, P27 found the goal setting helpful in establishing clarity, in keeping goals realistic, in finding ways to problem-solve goal barriers, and in motivating her to complete her goals:

That was the thing I found really useful so it was very good encouraging me to to set a very clear goal … set myself something very targeted. I tried my best not to do something too … too erratic cos I’d then never do it. But then having someone I was accountable to as well… and find ways made a ginormous difference.

P27

P44 found that the accountability from the peer facilitators helped her set goals that challenged her but were also achievable:

It was kind of like a nice way to stretch yourself a bit but also then get that that lovely reinforcement that the facilitators were giving kind of about how you were doing and so yeah the accountability was helpful.

P44

Being accountable to peers was something that some participants felt unsure about initially, but were more comfortable with as time went on:

I did set some goals … I found that a bit challenging from the sense of setting a goal and other people knowing what your goal is and that being kind of a public thing and I think my initial reaction was I don’t want to share a goal with anyone. I found that everyone was really supportive the facilitators were really good at encouraging you and like supporting you on the goals and it was nice to it was nice to be able to reflect back on it when it had gone well and when I hadn’t achieved the goal no-one was critical of it - they were quite encouraging.

P14

Participants reported finding the peer group delivery of the intervention helpful, with some caveats, most of which were related to the extent of information sharing and the level of participation and engagement across the group. For example, P14 was wary at first about sharing personal information in the group via her personal profile. As time passed and the group dynamics became clearer (and perhaps a sense of what data could be seen and by whom), she found interacting with other participants beneficial:

I think initially … I was quite daunted by doing something like on an open platform and where you were posting and other people could see what you were posting… but found it like easy to use and I think… as time went on I got less worried about posting on that like open forum and I found the interaction with other people helpful.

P14

P16 experienced the support of peers in the group as beneficial for managing negative emotions or discouragement, even when she was not logged in to the intervention:

Having that little group of cheer leaders and even though I only keep in contact with one of them in my mind sometimes if I’m having a negative moment or I’m having a bit of a waver I sometimes think of that group and I think come on you’ve got a little group of cheerleaders somewhere out there come on! (laughs) It’s something simple like that but it really does make a big impact. Especially on a negative day.

P16

The mental imagery of a supportive group of cheering peers was a resource P16 was still drawing on to help her 9-10 weeks after the intervention had finished.

The fact that peers shared personal information, experiences, goals, and progress was valued by participants, as it gave a sense of belonging to a group of relatable, recognizable, empathic humans, rather than interacting with an impersonal or “robotic” digital entity. For example, P21 contrasted taking part in the PCOS Hope intervention with using health apps:

Although I knew everything was very confidential it really … I felt it put a name to someone, so it wasn’t like (reads out number). When you were reading like feedback on somebody, or you could see little comments they were putting up it was like “That’s that girl” … and it became more personal for me anyway cos a lot of the time when you do things like this you feel like you’re just talking to robots. But it meant for me I was like this is actually really people that potentially know exactly how I’m feeling.

P21

Despite deriving benefits from seeing others share their personal information, some participants preferred to observe without sharing quite so much information themselves. For example, P18 described herself as lurking and seeing others’ goal progress:

There was no pressure to have to engage with that as well so sometimes I can be a bit of a lurker and see what other people were doing to complete goals etc.

P18

P18 described a sense that the platform exerted no pressure to engage or share personal information, although a (perhaps inevitable) consequence of this was that information sharing and group discussion varied across the group. For example, P18 found she did not have as much one-to-one interaction as she would have liked, and no geographically local connections were made:

I think the idea was that it was supposed to be a sort of quite a social thing as well. I didn’t quite have that experience. I’d comment on things on people’s posts, etc. but I thought there might be one to one messaging perhaps as someone, if I saw someone was more local had a similar experience. I understand probably quite a lot of us were quite shy so perhaps I could have been more out there with it. But there didn’t seem to be a lot of that in particular and I was kind of hoping for a little bit more of that.

P18

The lack of any pressure to engage also meant that information sharing and group discussion diminished in the later weeks of the intervention, something that was regretted by P14:

Towards the end of the course there was only there was only a small number it seemed that were active on there on the portal so it kind of would have been nice if there’d been a few more.

P14

#### Suggestions for Future Iterations of the Program

Future modifications to the program that were suggested by participants included introducing refined information-sharing options within the group, for example, offering the choice to share goals and progress with facilitators only, an option to access support from the peer facilitators for longer than the 6-week intervention period, and making the intervention more widely available as part of routine care for PCOS.

One participant suggested refining privacy settings to allow group members to share only some details of their goals with the peer group, or to share goals and goal progress anonymously:

Perhaps if there was an option for certain aspects of your goal setting to perhaps be anonymous. So it could maybe state that you had set a goal … but if you wanted the goal itself to be anonymous might have encouraged me a little bit more … or if you could like on the news feed it might appear that you had set a goal or something like that or you had completed it- that would still be able to be posted on but if it you know if you didn’t want to necessarily disclose the exact content.

P18

P14 would have liked access to the support of the peer facilitators after the 6-week intervention period, in order for participants who might have fallen behind the main group to catch up:

The facilitators were on the course for the six weeks and then once the six weeks were up they weren’t on the platform any more. And I think it may have been helpful to have I don’t know one more week when they were still available or a bit longer because if you weren’t able to keep up with getting through each week’s worth of stuff then by the time you got to the end not all of the support would be available.

P14

P44 suggested that the intervention should be made available for all newly diagnosed patients in the NHS:

To be honest its exactly the type of thing that the NHS should be rolling out for people who get a PCOS diagnosis because there’s so little support and if you’re if one of your goals isn’t fertility based there’s even less for you. Having something that people when they get that diagnosis can kind of go through and be like this is a serious condition it will have knock on effects on my life you know how can I best manage those? It is exactly the type of thing that I think should be rolling out.

P44

### Pre- and Postprogram Secondary Outcomes

Those participants who completed both the baseline and postprogram questionnaires were included in the pre- and postprogram quantitative analysis (n=11).

Median and IQRs for outcomes pre- and postintervention for participants who completed at least 1 session of the intervention and completed questionnaires at both T0 and T1 are shown below (Table 3).

Between T0 (baseline) and T1 (post intervention, 6 weeks), there were decreases in depression and anxiety, and increases in hope agency, hope pathways, and gratitude. There was a meaningful (≥3 points) increase in mental well-being.

In participants completing both baseline and follow-up measures, 73% (8/11) met clinical caseness for depression at T0 and 36% (4/11) at T1. In participants completing both baseline and follow-up measures, 45% (5/11) met clinical caseness for anxiety at T0 and 45% (5/11) at T1.

**Table 3:** Anxiety, depression, mental well-being, gratitude, hope agency, and hope pathways pre-and postintervention scores for adults with PCOS^a^ who accessed the online, 6-week, self-management intervention and completed follow-up measures in the multimethod proof-of-concept study.

Outcome	Preintervention (n=11), median (IQR)	Postintervention (n=11), median (IQR)
Depression (PHQ-9^b^)	11 (8-16)	7 (6-13)
Anxiety (GAD-7^c^)	7 (4-11)	6 (4-14)
Mental well-being (WEMWBS^d^)	40 (35-44)	48^e^ (40-54)
Gratitude (GQ-6^f^)	35 (27-37)	37 (32-39)
Hope agency (SHS^g^)	13 (10-15)	17 (12-19)
Hope pathways (SHS)	15 (14-17)	17 (12-20)

^a^PCOS: polycystic ovary syndrome.

^b^PHQ-9: Patient Health Questionnaire 9-Items.

^c^GAD-7: Generalized Anxiety Disorder 7-Items.

^d^WEMWBS: The Warwick-Edinburgh Mental Wellbeing Scale.

^e^Indicates a clinically meaningful change.

^f^GQ-6: Gratitude Questionnaire 6-Items.

^g^SHS: State Hope Scale.

## Discussion

### Overview

To the best of our knowledge, this is the first study to explore the feasibility, acceptability, and potential benefits of a PCOS self-management intervention combining positive psychology and cognitive behavioral therapy theory and practice. We aimed to test the intervention for its feasibility for web-based recruitment and delivery, and its acceptability to patients. It was feasible to recruit via the internet and deliver the intervention online. We also aimed to explore the intervention’s potential to improve anxiety, depression, and positive well-being.

### Principal Findings

We have explored proof of concept in that it was feasible to recruit via the internet and deliver this first web-based intervention for self-management support in PCOS. We have identified preliminary evidence that the intervention is acceptable to a range of participants. Women who completed the program and reported their experiences described it as acceptable and beneficial, highlighting perceived gains in self-care, goal-setting, mental health, self-compassion, openness about PCOS, and preparedness for future health needs. They reported valuing concise content, practical tools, gratitude practices, and peer support, which enhanced motivation, confidence, and sustained behavioral change. Suggestions for improvement included more flexible goal-sharing options, extended facilitator support, and better integration into NHS care pathways.

This study produced encouraging data on the primary outcomes of adherence and engagement, with 46% of those using the intervention meeting the adherence definition and 29% completing the whole program. This level of adherence is similar to that typically seen in digital mental health interventions [46].

Descriptive quantitative analysis suggests the intervention has potential benefits for anxiety, depression, and positive well-being. Those who were followed up showed, on average, decreased scores on depression and anxiety, and significantly increased scores on hope agency, hope pathways, and gratitude between pre- and postintervention time points. There were meaningful increases in positive mental well-being between pre- and postintervention time points.

### Limitations

While we were able to successfully recruit from social media with the support of the patient advocacy organization, there is potential for selection bias in our sample. For example, women with PCOS who are already following a patient advocacy organization on social media may have accessed the organization’s educational materials and be better informed about their condition, or they may be more highly motivated to take part in research or seek self-management support. They may not be representative of the demographic characteristics of all adults living with PCOS, which we know may vary across age, ethnicity [60], and gender [61]. For future studies, attention should be given to recruiting from a wider range of sources and to monitoring key characteristics of participants recruited, including, for example, PCOS phenotypes or symptom clusters, ethnicity, gender, and socioeconomic status. As this was a proof-of-concept study, limited demographic data (age and time since diagnosis only) were collected, and no information was recorded on prior experience with therapy or digital self-management programs. More detailed demographic data and information on participants’ experience will be collected at baseline in future trials.

Although informed by a systematic review of adherence in digital mental health interventions [46], the adherence definition we chose, of at least 3 sessions, was selected somewhat arbitrarily, as there is no agreed-upon definition of adherence. We cannot draw clear conclusions as to how many sessions of the intervention are required to achieve improvements in the secondary outcome measures, and it is possible that some intervention sessions are redundant, or that participants become fatigued after a certain number of sessions. A future, fully powered study may enable us to identify the number of intervention sessions required to achieve clinically and statistically significant improvements in the secondary outcome measures.

There may be some selection bias in the process by which participants volunteered for the qualitative interviews we used to assess acceptability. For example, participants who found the program particularly beneficial may have been more likely to volunteer for an interview. Critical feedback from 1 participant interviewed suggested she experienced peer interaction in the program as nonsupportive or, at times, counterproductive. The potential for a negative impact from peer interaction should be investigated further in future studies. Individuals who are less engaged with an intervention are less inclined to participate in qualitative research with volunteer samples. This might be addressed in future work by including exit surveys for participants who drop out, in order to identify barriers to engagement or purposively recruiting intervention noncompleters for interview and emphasizing to them that their perspectives are valuable to the researchers, particularly if they are critical.

We considered a range of outcomes, including those measuring the adverse impact of PCOS on health-related quality of life, for example, the Polycystic Ovary Syndrome Questionnaire [62] and the Modified Polycystic Ovary Syndrome Questionnaire [63]. As we were interested in the potential for enhancing positive well-being, we chose WEMWBS as a measure of positive well-being. Future projects will test the feasibility of using measures that address PCOS-specific concerns. We conducted the GAD-7, PHQ-9, and WEMWBS assessments at 2 points—directly before and after the 6-week intervention—using the standard 2-week recall timeframe to maintain the measures’ recognized reliability and validity. This approach allowed us to capture participants’ recent levels of anxiety, depression, and well-being, as well as any overall changes, while minimizing burden and safeguarding data quality. Nonetheless, future studies could explore a more detailed picture of session-by-session changes by incorporating more frequent assessments.

As this was an exploratory proof-of-concept study, we intentionally adopted a purely qualitative approach to explore the variety and complexity of perceived physical health changes, which may be assessed more robustly with quantitative or self-reported biomarker measures in future studies.

More work could be carried out to enhance adherence and engagement with the intervention by building on participant feedback on acceptability. For example, intervention content could be updated and expanded to include newly emerging scientific information on PCOS and information of interest to older and longer diagnosed participants, offering a range of privacy settings that allow participants to select which peers see particular posts, and including more prompts to encourage peer interaction. As this was a descriptive exploratory study, we did not attempt to mitigate or measure the influence of peer effects or peer dynamics. The course facilitators encouraged peer interaction, for example, by encouraging participants to post questions on discussion forums and respond to peers’ posts, but this was not compulsory. Future research will explore preferences for and experience and effects of peer interaction.

The follow-up rate for participants completing T1 postintervention outcome measures was low, with 11/48 (23%) completing these. This means that the sample for whom follow-up data is available is not likely to be representative. For example, follow-up data is only available for women diagnosed between 3 and 25 years ago. In future studies, it may be advisable to offer compensation to study participants to increase the number completing follow-up measures. It would also be insightful to follow up with participants longer term to assess any prolonged impact on anxiety, depression, and well-being.

This proof-of-concept study had no control group and was not powered to detect statistically significant differences in pre- and postprogram scores on secondary outcomes. Future feasibility trials will be required to confirm the sample size required for a definitive trial. Therefore, while we have established proof of concept, we are unable to demonstrate definitively that the intervention is effective. Data from a fully powered trial with an adequate sample size and an appropriate control arm will allow us to report any statistically significant differences in pre- and postprogram scores for both an intervention group and a control group and demonstrate efficacy under trial conditions. A fully powered study would also help us identify the characteristics of participants more likely to fully engage with and benefit from the intervention.

### Conclusions

We have established proof of concept for the Hope PCOS intervention. The next challenges for our team will be to conduct definitive trials of our intervention in a range of clinical settings, to test its clinical and cost-effectiveness, and to find ways to integrate it into existing clinical care pathways for PCOS. If successful, H4C’s platform and the Hope PCOS intervention may support the development of prototype interventions for other women’s health conditions.

## Appendix

Multimedia Appendix 1 CONSORT 2010 checklist.

Multimedia Appendix 2 Intervention content.

Multimedia Appendix 3 TIDieR checklist.

Multimedia Appendix 4 COREQ checklist.

## Abbreviations

AFAB: assigned female at birth
CONSORT: Consolidated Standards of Reporting Trials
COREQ: Consolidated Criteria for Reporting Qualitative Studies
GAD-7: Generalized Anxiety Disorder 7-Items
GQ-6: Gratitude Questionnaire
H4C: Hope for the Community
ISO: International Organization for Standardization
NHS: National Health Service
ORCHA: Organisation for the Review of Care and Health Apps
PCOS: polycystic ovary syndrome
PHQ-9: Patient Health Questionnaire 9-Items
SHS: State Hope Scale
TIDieR: Template for Intervention Description and Replication
WEMWBS: The Warwick-Edinburgh Mental Wellbeing Scale

## Data Availability

The quantitative datasets generated or analyzed during this study are available from the corresponding author on reasonable request.
