# Supplementary material for: Exploring Proof of Concept for a Novel Web-Based Self-Management Support Intervention for Polycystic Ovary Syndrome: Multimethod Study
Source: JMIR Form Res. 2026 Feb 17;10:e69206. doi: 10.2196/69206 (PMC12957944; doi:10.2196/69206)
Supplement: Multimedia Appendix 2 [file formative_v10i1e69206_app2.pdf]

Hope PCOS[a] intervention session content for online, 6-week self-management intervention for adults with PCOS in a multi-method proof of concept study.

Week	Session title and content
**1**	**HOPE for PCOS**
	Welcome/introductions<br>Responsibilities/ground rules<br>Hope for Your PCOS journey<br>Support from health professionals, peers and (UK PCOS charity)<br>PCOS Basics: Information about PCOS signs and symptoms<br>Androgen excess & insulin resistance<br>Test your PCOS basics: quiz<br>Self-management for PCOS – you don't have to do this alone<br>Relaxation breathing<br>Self-compassion<br>Gratitude diary<br>Goal setting<br>Open space forum<br>Useful resources
**2**	**Managing the stress of PCOS**
	Solution focused goal feedback<br>Managing stress<br>Mindfulness<br>Physical activity for stress management - get active, feel good<br>Managing common unhelpful thinking patterns (CBT[b])<br>Mindfulness - and am I doing this 'right'?<br>Why self-compassionate mindfulness?<br>Compassion-focused therapy<br>Gratitude diary<br>Goal setting<br>Open space forum<br>Useful resources
**3**	**Feeding your mind & body well**
	Solution focused goal feedback<br>Eating well in PCOS - role of food intake, blood sugar and insulin<br>Why being insulin resistant is a problem for the whole body<br>Why it may sometimes sound like health professionals are being preachy or<br>Eating well without depriving yourself<br>Losing weight in PCOS<br>Eating mindfully to eat well in PCOS<br>Over-eating and binge eating in PCOS<br>Some helpful tips for reducing the chances of over-eating<br>Feeding your mind and body well<br>Feeding your mind: three systems regulating our emotions<br>Ways to soothe yourself without over- or under-eating<br>Getting to know your inner critic<br>Developing a compassionate ideal<br>Gratitude diary<br>Goal setting

	Open space forum<br>Useful resources
**4**	**Body image, intimacy & close relationships**
	Solution focused goal feedback<br>Common body changes in PCOS<br>Difficult emotions that can come with body changes<br>Getting ready to be self-compassionate<br>Getting ready to be self-compassionate towards feelings of embarrassment or shame<br>Compassion for your perceived flaws<br>Body changes, sexuality & intimacy<br>Responding to change: acceptance, treatments and adaptations<br>Overcoming embarrassment to get help for body changes<br>Treatments<br>Gratitude diary<br>Goal setting<br>Open space forum<br>Useful resources
**5**	**Staying healthy with PCOS**
	Solution focused goal feedback<br>Focusing on your future health<br>Ways to manage emerging health concerns and worries about your future health<br>Getting peer support<br>Maximising your psychological resources<br>Maximise the support you get from health services and health professionals<br>Healthcare specialists who treat PCOS<br>Activity: Summarising concerns and requesting a referral<br>Communicating clearly and assertively with health professionals<br>Activity: Shared agenda setting at healthcare appointments<br>When communicating clearly and asking assertively don't get you what you want – community support and activism<br>Gratitude diary<br>Goal setting<br>Open space forum<br>Useful resources
**6**	**Keeping PCOS in its place – your strengths and life goals**
	Solution focused goal feedback<br>Character strengths<br>Life priorities<br>Motivational imagery<br>Sharing our successes<br>Self-compassion<br>Activity: self-compassionate letter<br>Gratitude diary<br>Goal setting<br>Open space forum<br>Further Resources and further reading<br>Signposting to ongoing participation with PCOS related groups and networks

[a]PCOS: polycystic ovary syndrome.

[b]CBT: cognitive behavioral therapy.
