# Supplementary material for: Exploring Proof of Concept for a Novel Web-Based Self-Management Support Intervention for Polycystic Ovary Syndrome: Multimethod Study
Source: JMIR Form Res. 2026 Feb 17;10:e69206. doi: 10.2196/69206 (PMC12957944; doi:10.2196/69206)
Supplement: Multimedia Appendix 3 [file formative_v10i1e69206_app3.pdf]

The TIDieR (Template for Intervention Description and Replication) Checklist*:

Information to include when describing an intervention and the location of the information

Item number	Item	Where located **	
		Primary paper (page or appendix number)	Other † (details)
1.	**BRIEF NAME**<br>***Hope PCOS***	1,4,5	______________
	**WHY**		
2.	*Co-designed for PCOS women and others AFAB to address self-management, psychological well-being, and social support needs. Grounded in positive psychology, positing that meeting information needs, while cultivating hope and gratitude fosters emotional self-regulation and resilience, thereby empowering individuals with PCOS to improve psychological well-being, cope adaptively, and manage depression and anxiety through enhanced positive affect and goal-directed behaviour.*	4,5,6	______________
	**WHAT**		
3.	*Web-based programme, interactive materials, gratitude diary, cognitive behavioural educational modules, peer support, secure forum* [*www.h4c.org.uk*](http://www.h4c.org.uk)	4,5,6	______________
4.	*6-week, asynchronous with weekly themed modules, peer feedback, goal setting, online discussion*	4,5,6	______________
	**WHO PROVIDED**		
5.	*Delivered by trained peer facilitators with lived PCOS experience*	4	______________
	**HOW**		
6.	*Online, web-based, asynchronous, group format*	4	______________
	**WHERE**		
7.	*Online secure platform*	4	______________

	**WHEN and HOW MUCH**		
**8.**	*6 weekly modules; content released weekly; approximately 2.5 hours per module but users access at their pace*	4	______________
	**TAILORING**		
**9.**	*Open goal setting, individual support via forum, open forum for participant engagement*	4	______________
	**MODIFICATIONS**		
**10.‡**	*None made in this first proof-of-concept study; refinements planned for subsequent trials*	N/A	______________
	**HOW WELL**		
**11.**	*Module release schedules monitored, peer facilitator checklist, engagement metrics collected*	4, 5	______________
**12.‡**	*Engagement/adherence data: sessions completed, gratitude diary entries, qualitative exit interviews on experience*	12-23	______________

** **Authors** - use N/A if an item is not applicable for the intervention being described. **Reviewers** – use '?' if information about the element is not reported/not sufficiently reported.

† If the information is not provided in the primary paper, give details of where this information is available. This may include locations such as a published protocol or other published papers (provide citation details) or a website (provide the URL).

‡ If completing the TIDieR checklist for a protocol, these items are not relevant to the protocol and cannot be described until the study is complete.

* We strongly recommend using this checklist in conjunction with the TIDieR guide (see *BMJ* 2014;348:g1687) which contains an explanation and elaboration for each item.

* The focus of TIDieR is on reporting details of the intervention elements (and where relevant, comparison elements) of a study. Other elements and methodological features of studies are covered by other reporting statements and checklists and have not been duplicated as part of the TIDieR checklist. When a **randomised trial** is being reported, the TIDieR checklist should be used in conjunction with the CONSORT statement (see www.consort-statement.org) as an extension of **Item 5 of the CONSORT 2010 Statement.** When a **clinical trial protocol** is being reported, the TIDieR checklist should be used in conjunction with the SPIRIT statement as an extension of **Item 11 of the SPIRIT 2013 Statement** (see www.spirit-statement.org). For alternate study designs, TIDieR can be used in conjunction with the appropriate checklist for that study design (see www.equator-network.org).
