# Supplementary material for: Exploring Proof of Concept for a Novel Web-Based Self-Management Support Intervention for Polycystic Ovary Syndrome: Multimethod Study
Source: JMIR Form Res. 2026 Feb 17;10:e69206. doi: 10.2196/69206 (PMC12957944; doi:10.2196/69206)
Supplement: Multimedia Appendix 4 [file formative_v10i1e69206_app4.pdf]

Consolidated criteria for reporting qualitative studies (COREQ): 32-item checklist

No. Item	Guide questions/description	Reported on Page #
**Domain 1: Research team and reflexivity**		
Personal Characteristics		
1. Interviewer/facilitator	Which author/s conducted the interview or focus group?<br>*The interviews were conducted by a quality assurance manager working for H4C*	7
2. Credentials	What were the researcher's credentials? E.g. PhD, MD<br>The interviewer had a BSc. and PhD.<br>*Deductive thematic analysis was done by the interviewer using the audio recordings and checked by the first author (Carol Percy, BSc. PhD.)*<br>*The reflexive thematic analysis was conducted by CP and discussed with second author (Andy Turner, BSc., PhD.).*	7-8
3. Occupation	What was their occupation at the time of the study?<br>*BA was a quality assurance manager.*<br>*CP was a university lecturer*<br>*AT a university professor*	7-8
4. Gender	Was the researcher male or female?<br>*The interviewer was female*	7
5. Experience and training	What experience or training did the researcher have?<br>*The interviewer had previous experience in qualitative interviewing and extensive experience in working with people living with long-term health conditions.*<br>*CP was an experienced qualitative researcher with lived experience of PCOS.*	7
Relationship with participants	*The interviewer was not known to the participants prior to interview.*	7
6. Relationship established	Was a relationship established prior to study commencement?	N/A

7. Participant knowledge of the interviewer	What did the participants know about the researcher? e.g. personal goals, reasons for doing the research<br>*Interviewer introduced herself at beginning of the interviews, explained her role (worked for H4C) and purpose of the research (to explore participants' experiences of and views on how to improve the Hope PCOS course).*	7
8. Interviewer characteristics	What characteristics were reported about the interviewer/facilitator? e.g. Bias, assumptions, reasons and interests in the research topic.<br>*The interviewer was neither part of the intervention development team nor directly involved in the delivery of the intervention. As quality assurance lead she was chosen to conduct the interviews to encourage open and honest disclosure from participants.*	7
**Domain 2: study design**		
Theoretical framework		
9. Methodological orientation and Theory	What methodological orientation was stated to underpin the study? e.g. grounded theory, discourse analysis, ethnography, phenomenology, content analysis<br>*The study used a mixed inductive–deductive, realist, experiential framework, with thematic analysis conducted following Braun and Clarke's flexible, pragmatic approach. Theme development was iterative and reflexive, balancing researcher subjectivity with the need to rapidly inform the intervention team of potential platform and material improvements.*	8
Participant selection		
10. Sampling	How were participants selected? e.g. purposive, convenience, consecutive, snowball<br>*Purposive sampling was used for exit interviews with intervention 'adherents'. Of the 22 women who had completed at least 3 hope sessions and were invited to exit interview, 8 agreed and were interviewed.*	7

11. Method of approach	How were participants approached? e.g. face-to-face, telephone, mail, email<br>*Email with follow-up telephone call to agree time and date.*	7
12. Sample size	How many participants were in the study?<br>*Eight were interviewed*	7
13. Non-participation	How many people refused to participate or dropped out? Reasons?<br>*Participation was voluntary opt in by email reply. No refusals* per se. *All who repsonded to the email invitation were interviewed.*	7
Setting		
14. Setting of data collection	Where was the data collected? e.g. home, clinic, workplace<br>*By telephone*	7
15. Presence of non-participants	Was anyone else present besides the participants and researchers?<br>*No-one.*	7
16. Description of sample	What are the important characteristics of the sample? e.g. demographic data, date<br>*Age and time since diagnosis were recorded.*	11
Data collection		
17. Interview guide	Were questions, prompts, guides provided by the authors? Was it pilot tested?<br>*Interview questions included expectations and experiences of the programme and any changes in mental or physical health or self-management activities attributed to participation in the programme. Not piloted as these had been used in previous research.*	7
18. Repeat interviews	Were repeat interviews carried out? If yes, how many?<br>*No repeat interviews were carried out.*	7
19. Audio/visual recording	Did the research use audio or visual recording to collect the data?<br>*Audio recording was used.*	7
20. Field notes	Were field notes made during and/or after the interview or focus group?<br>No	N/A
21. Duration	What was the duration of the interviews or focus group?	7

	30-60 minutes	
22. Data saturation	Was data saturation discussed?<br>*Data saturation was not used. For reflexive TA, data saturation is not viewed as a meaningful or appropriate standard, and claims to have "reached saturation" are discouraged in favour of transparency about the reasoning behind sample size and the depth of analysis.*	8
23. Transcripts returned	Were transcripts returned to participants for comment and/or correction?<br>*As this was an unfunded study without compensation for participation, to avoid burdening participants member checking of transcripts was not used.*	8
**Domain 3: analysis and findings**		
Data analysis		
24. Number of data coders	How many data coders coded the data?<br>*Two: RA did deductive coding, CP did inductive coding and reflexive thematic analysis.*	8
25. Description of the coding tree	Did authors provide a description of the coding tree?<br>*No coding tree used. Braun and Clarke's reflexive thematic analysis supports flexible, iterative coding and theme development, and does not rely on formal coding trees as used in some other qualitative analytic traditions.*	7
26. Derivation of themes	Were themes identified in advance or derived from the data?<br>*Two deductive themes (domain summaries) were purposively identified in advance: 'Experience of content, tools and format'; and 'Suggestions for future iterations of the programme'. The minor themes within these were derived inductively. Nine reflexive thematic analysis themes were derived by inductive coding following Brain & Clarke's recommendations.*	7-8

27. Software	What software, if applicable, was used to manage the data?<br>*An Excel spreadsheet was used to sort codes and data extracts iteratively in order to identify themes.*	8
28. Participant checking	Did participants provide feedback on the findings?<br>*As this was an unfunded study without compensation for participation, to avoid burdening participants member checking of analysis was not used.*	8
Reporting		
29. Quotations presented	Were participant quotations presented to illustrate the themes/findings? Was each quotation identified? e.g. participant number<br>*Quotations are used and these are identified by participant number.*	13-23
30. Data and findings consistent	Was there consistency between the data presented and the findings?<br>*We believe the data presented are consistent with the findings reported.*	13-24
31. Clarity of major themes	Were major themes clearly presented in the findings?<br>Major themes are identified in a table and supported with participant quotations.	13
32. Clarity of minor themes	Is there a description of diverse cases or discussion of minor themes?<br>*Minor themes are discussed with participant quotations and some variation is discussed, e.g. while most participants found the intervention content personally relevant not all did; most found the peer interaction helpful but some less so.*	13-24
